# The Mediating Effect of Eating Behaviors on Interoception, Self-Regulation and Weight Status Among College Students

**DOI:** 10.3390/nu16233986

**Published:** 2024-11-21

**Authors:** Shanté Jeune, Paulo Graziano, Adriana Campa, Catherine Coccia

**Affiliations:** 1 Department of Health Sciences, University of Central Florida, Orlando, FL 32816, USA; 2 Department of Psychology, Florida International University, Miami, FL 33199, USA; pgrazian@fiu.edu; 3 Department of Dietetics and Nutrition, Florida International University, Miami, FL 33199, USA; campaa@fiu.edu (A.C.); ccoccia@fiu.edu (C.C.)

**Keywords:** interoception, self-regulation, eating behaviors, mediation, college students

## Abstract

Background/Objectives: Obesity among college students has been consistently high in the recent decades. Regulatory processes such as interoception and self-regulation have been studied to identify specific health behaviors that lead to weight gain. Reduced interoception and self-regulation may lead to increased body mass index (BMI), however, various eating styles may indirectly affect this relationship. It is proposed that poor interoception and self-regulation can increase the incidence of maladaptive eating styles, such as emotional or external eating, which may indirectly contribute to weight gain. Conversely, eating styles like cognitive restraint and intuitive eating may indicate an opposing indirect effect, exhibiting eating behaviors likely to maintain optimal weight status. To date, it is unknown which eating styles mediate the relationship between interoception and self-regulation on BMI. Additionally, study variables were examined throughout time to identify any potential influences throughout a typical semester. Methods: There were 104 female participants who completed the study measures. Participants were primarily identified as Hispanic (75.1%), mean age = 23.39 (SD = 6.312), and mean BMI = 25.45 (SD = 5.48) at baseline. Preliminary statistics and longitudinal mediation analyses were conducted to examine the relationships among eating styles, interoception, self-regulation, and BMI. Results: Intuitive eating was the only eating style that was found to be a significant mediator among both interoception and self-regulation, and BMI. It is theorized that these regulation skills occur more commonly among college females who intuitively eat, thus accurately explaining the associations with BMI. Conclusions: The study has provided foundational evidence on the indirect effect of eating behaviors on one’s relationship with interoception and self-regulation on BMI and can be useful in future interventions regarding college students and their associated risk for obesity.

## 1. Introduction

Obesity rates among college students have been a major topic of concern for several years. In 2021, approximately 38% of college students were classified as overweight or obese [1]. Issues that commonly stem from excessive weight gain and obesity include chronic diseases such as cardiovascular disease and diabetes, and increased mortality risk later in adulthood [2]. Since college is often considered the first time that many young adults make their own food choices and form long-term eating patterns, this timeframe is considered highly influential on one’s risk for obesity later in life [3]. Internal regulation skills, like interoception and self-regulation, have recently gained attention in obesity research due to their relationship with one’s eating behaviors and, ultimately, weight status.

### 1.1. Internal Regulation Skills

Interoception is the perception of one’s internal awareness and responsiveness to bodily signals [4]. It is often characterized as the communicative link between the body and the brain, which may serve to regulate one’s hunger and satiety needs [5,6,7]. Innately, interoceptive processes are highly sensitive and responsive to bodily systems to make constant adjustments for successful homeostasis. However, interoception can become reduced or blocked due to external factors such as stress or poor emotional regulation. Sensitivity to interoceptive awareness differs vastly between person to person. Nevertheless, there are strong correlations between interoception and eating behavior outcomes. For example, individuals who had increased internal awareness were more likely to participate in healthy eating behaviors and lifestyle choices [8,9].

Self-regulation represents the active monitoring of one’s physiological, emotional, and attentional needs to achieve a specific goal and can be dependent on one’s level of interoceptive awareness [10,11,12]. Interoception serves as a prerequisite for self-regulation, as interoception allows the individual to become aware of the internal cues to advance the action of self-regulation. For example, active self-regulation can strengthen the practice of consuming food when experiencing feelings of hunger and stopping intake when full. Both internal regulation skills have been commonly associated with healthier eating styles, which may lead to weight management in college students over time [13,14,15,16]. However, it is still unclear how one’s eating style may serve as a mediating variable on the relationship between a person’s internal regulation skills and their weight status.

### 1.2. Eating Styles

In college students, differing levels of internal regulation skills may lead to various eating behavior patterns over time. Non-purposeful eating behaviors include emotional, external, and uncontrolled eating styles whereas purposeful eating behaviors include cognitive restraint and intuitive eating styles [11]. Emotional eating is generally defined as eating in response to either positive or negative emotional cues as a coping mechanism [17,18]. Poor interoception was linked to increased emotional eating among college females and adults living with obesity [16,19]. Additionally, specific facets of self-regulation, such as emotional regulation, indicated increased difficulties related to emotional eating in female college students [20]. External eating is eating in response to an external food-related stimulus like the sight or smell of food, regardless of having feelings of hunger or satiety [18]. Reduced conscientiousness (interoception) and self-regulation overeating habits have been shown among external eaters, thus causing external eaters to consume more energy dense food items [21,22]. Uncontrolled eating, described as the loss of control while consuming food, is viewed more so as a continuum that starts from eating impulsivity to more severe overeating behaviors [23]. In this eating style, an individual is more sensitive and reactive to external food cues as the result of all interoceptive and self-regulative processes being reduced.

Alternatively, college students with increased interoception and self-regulation may become more purposeful in their eating habits. Purposeful eating is an umbrella term utilized to describe one’s level of intentional behavior that determines whether to eat or not. Purposeful eating behaviors consist of cognitive restraint and intuitive eating styles [11]. Both eating styles require higher self-awareness and control that most often correlate with health eating behavior outcomes. Cognitive restraint is most similar to traditional dieting, where a person is highly aware of their internal signals yet override hunger cues to purposefully restrict one’s intake [24]. Results indicating cognitive restraint eating as a healthy eating style have been mixed. However, previous evidence notes that individuals who are successful in practicing cognitive restraint exhibit other healthy eating practices and reduced their obesity risk, compared to those who are unsuccessful [25,26]. Additionally, those who were able to restrict their intake were correlated with lower cravings scores, compared to those who attempted to diet [27]. Despite mixed outcomes in eating, previous evidence indicates that interoceptive responsiveness was significantly associated with cognitive restraint [16]. Intuitive eating is the non-diet approach that is centered around the individual’s internal body signaling response such as their hunger and satiety cues [28,29]. To effectively practice intuitive eating, individuals must be able to accurately identify and respond to their internal hunger cues via interoception and regulate their intake based on the guided cues [28,30,31].

**Figure 1:**
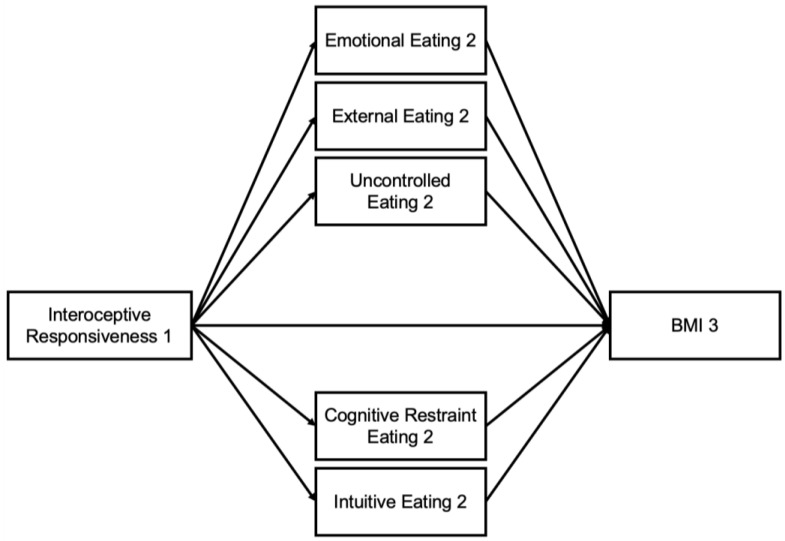
Mediation Model with Interoceptive Responsiveness and BMI through Eating Behaviors. Note: BMI = Body Mass Index.

**Figure 2:**
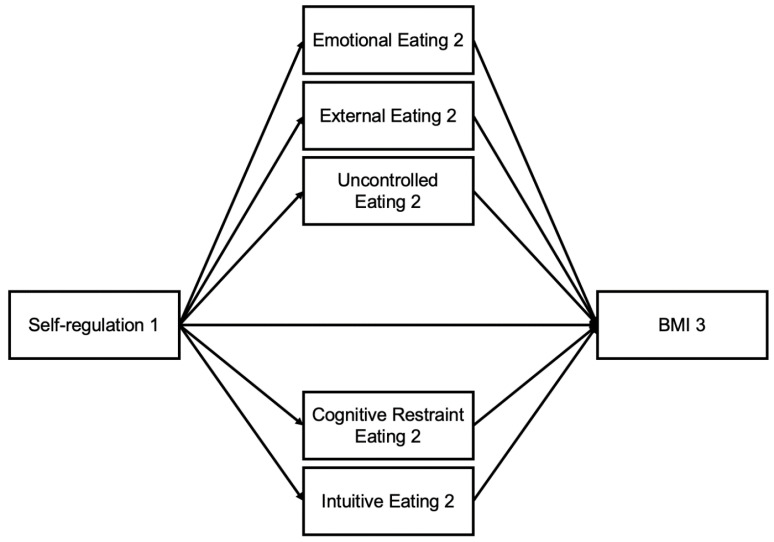
Mediation Model with Self-regulation and BMI through Eating Behaviors.

It is theorized that increased interoception and self-regulation can effectively maintain healthy body mass index (BMI), when individuals have more purposeful eating behaviors. Inversely, it is suggested that reduced interoception and self-regulation leads to increased BMI among those with non-purposeful eating behaviors. Currently, it is unknown which eating styles mediate the associations of interoception and self-regulation on BMI. In utilizing the 5 most common eating styles, it is important to determine which eating style most prominently explains the association of interoception and self-regulation on BMI. Taken one step further, the concept of time is also considered. Specifically in college students, assessing the mediating role of eating behaviors on the associations of interoception and self-regulation on BMI at three separate timepoints (beginning, mid-point, and end of the semester) can provoke a more detailed conversation. To determine the mechanistic effects between the variables, examining each variable at a different timepoint can display the potential influence that one variable may have on another throughout time. It is necessary to examine internal regulation skills at the beginning of the semester (Timepoint 1 [T1]) prior to any potential conflicting external factors that tend to happen within the term. Eating styles were assessed at Timepoint 2 (T2) to understand the students’ typical behavior during the school term. Lastly, to determine the outcome effects by the end of one’s term, BMI was assessed at Timepoint 3 (T3) to determine potential directionality of the associations. Taken together, the purpose of this study is to examine the indirect effects of multiple eating styles on the associations of interoception and self-regulation on BMI. We predict that individuals with increased interoception and self-regulation will have decreased BMI through use of purposeful eating behaviors (cognitive restraint/intuitive eating). We also predict that individuals with reduced interoception and self-regulation will have increased BMI through the use of non-purposeful eating behaviors (emotional, external, uncontrolled eating) (Figure 1 and Figure 2).

## 2. Methods

### 2.1. Participant Recruitment and Procedures

Participants were primarily recruited through the university’s psychology research participation pool online system, SONA, at a 4-year large, metropolitan university in Southern Florida. Other recruitment methods included classroom announcements and recruitment flyers. The inclusion criteria were undergraduate male and female students, aged 18 years or older. The exclusion criteria for this study consisted of taking medications that altered appetite, previously diagnosed mood or eating disorders, pregnant or planning to become pregnant, and/or student athletes. Interested students completed an eligibility survey via Qualtrics to determine eligibility. Students eligible to participate received an online informed consent to complete before the baseline surveys. At all 3 timepoints (baseline, 1-month follow-up, and 2-month follow-up), participants provided self-reported demographic and anthropometric information and completed measures using validated surveys via Qualtrics. Study completion took approximately 30 min at each timepoint. Participants who completed all timepoint measures received a $15 Amazon gift card. Study protocols were reviewed and approved by the university’s Institutional Review Board (IRB-02-0556). The IRB approval dates were 12 November 2020–12 November 2023.

Study data were previously collected from a repeated-measures, observational research study with a total of 279 participants. The previous study utilized the G*power software (version 3.1.9.7) to determine sample size estimation using multiple linear regression analysis (primary analysis) with an effect size of 0.20, power analysis of 80% and associated attrition rates (35%) to achieve a minimum sample size of 91 participants by the end of the study (reference blinded for review). For this study, ad-hoc power/effect size calculations were performed to confirm sufficient sample size for the current study’s primary analysis.

At the end of the study, there were only 13 males who participated in the study. Because the number of male participants was low, the researchers of this study excluded the male participants (*n* = 13) during data analysis process due to the inability to conduct gender-stratified analyses previously found in this research area. Additionally, male participants would be underrepresented in overall analyses, thus, effective conclusions for this subpopulation cannot be determined. After participant drop-out and the removal of male participants (*n* = 13), there were 229 (82% of recruited participants) females who completed baseline measures. At the end of the study (T3), there were 104 females that completed all study measures (45% retention within study).

### 2.2. Measures

#### 2.2.1. Interoception

Interoceptive responsiveness, defined as the response to one’s internal signaling, was measured by the Body Responsiveness Scale (BRS). The BRS is a 7-item scale that measures how an individual responds to their bodily sensations [28]. Responses were based on a 7-point Likert scale indicating 1 as ‘not at all true’ and 7 as ‘always true of me’. Increased scoring demonstrates increased interoceptive responsiveness. BRS has demonstrated evidence of good internal consistency (α = 0.83) and convergent validity among adult men and women [32,33]. In our study, BRS demonstrated good reliability with a Cronbach’s alpha (α) of 0.75 for T1.

#### 2.2.2. Self-Regulation

Self-regulation was measured via the Self-Regulation of Eating Behavior Questionnaire (SREBQ). The SREBQ is a 5-item questionnaire that assesses an individual’s self-regulation capacity [34]. A Likert scoring system of 1 (Never) to 5 (Always) was used. Good internal consistency (α = 0.75) and construct validity, with tests of concurrent, convergent and discriminant validity, was reported in the general adult (20–65 years) population [34]. In our study, the questionnaire’s internal consistency was α = 0.69 for T1.

#### 2.2.3. Eating Behaviors

Eating behaviors were measured using the following 3 validated questionnaires: Dutch Eating Behavior Questionnaire (DEBQ), Three-factor Eating Questionnaire (TFEQ-R18), and Intuitive Eating Scale-2 (IES-2). The DEBQ is a 33-item questionnaire that contains three subscales of Emotional eating, External eating, and Restrained eating [18]. The assessment indicates a person’s eating behavior based on three main psychological theories [18]. For this study, the researchers only utilized the Emotional eating and External eating subscales. Items were scored on a Likert scale ranging from 1 (seldom) to 5 (very often). The DEBQ has maintained good internal consistency and construct validity, assessed with exploratory and confirmatory factor analyses, since its development [18,35,36,37,38]. In our study, DEBQ had high internal consistencies for Emotional eating (α = 0.96) and for External Eating (α = 0.86) in T2.

The TFEQ-R18 is an 18-item assessment that measures eating behavior concepts of Cognitive Restraint, Uncontrolled and Emotional eating [24]. The Cognitive Restraint and Uncontrolled eating subscales were only utilized in this study. Responses were scored using a four-point scale of 1–4, with the higher values signifying increased behavior. Demonstration of good internal consistency (cognitive restraint α = 0.76 and uncontrolled α = 0.86) and construct validity was found in a similar sample of young adults [24]. In our sample, TFEQ-R18 had Cronbach’s alpha (α) of 0.68 for Uncontrolled eating and 0.76 for Cognitive Restraint Eating at T2, indicating good reliability.

The IES-2 is a 23-item questionnaire that assesses the individual’s ability to adhere to their internal hunger and satiety cues, regarding when to eat [39]. The IES-2 was scored using a Likert scale of 1 (strongly disagree) to 5 (strongly agree). In college females, the IES-2 had good construct validity and reliability reported [39]. In the current study, the scale had an internal consistency of α = 0.87 for T2, representing good reliability.

#### 2.2.4. Weight Status

Participant weight was provided via self-report via Qualtrics survey. At baseline, participants received an online study manual and were instructed on how to record their height and weight using a digital scale and tape measure. At each timepoint, participants reported their weight measurements while completing the other questionnaires. BMI was calculated using the individual’s baseline height and weight at each timepoint using the formula [weight (kg)/[height (m)]^2^ [40]. For demographics, BMI were classified into 4 categories using the following Center for Disease Control guidelines: Underweight (BMI < 18.5), Healthy weight (18.5–24.9), Overweight (25.0–29.9), and Obesity (BMI ≥ 30.0) [40]. However, BMI was assessed continuously in mediation analyses.

#### 2.2.5. Statistical Analysis

Data were analyzed using SPSS Statistics v29.0 for descriptive, assumptions, and preliminary/main analyses. Also, ad-hoc effect size (using Cohen’s d) and power analyses were conducted to ensure the current study sample size was suitable for main analyses [41,42]. General assumptions were met to test for mediation. Study variables were using a continuous scale and data were distributed normally [43]. Standardized residuals were examined for any absolute values greater than 3 [44]. Mediation analyses were conducted using the recommended PROCESS v4.2 system by Andrew Hayes [45]. First, path analysis using maximum likelihood estimation to examine direct associations between the variables were assessed. Then, both total and specific indirect effects were examined to assess potential mediation. A significance level of *p* ≤ 0.05 was used for the direct effect analyses. To signify indirect effect significance, the researchers performed the percentile bootstrapping method. We utilized 5000 samples with a 95% confidence interval to detect potential significance. Complete data are required for the PROCESS v4.2 system. Casewise deletion was automatically applied when using the PROCESS v4.2 system to conduct mediation analysis. The outcome data were assessed, and missing data were primarily attributed from the dropout between each of the timepoints. Missing data were not due to any of the observed or unobserved variables in the study and considered “missing completely at random” (MCAR), allowing for reduced potential biases in our analyses [46]. Therefore, only participants who completed all study measures at each timepoint (*n* = 104) were included in the study’s analyses.

## 3. Results

### 3.1. Participant Characteristics

Participants were predominantly identified as Hispanic (75.1%) with an average age of 23.39 (SD = 6.312) and BMI average of 25.45 (SD = 5.48) at baseline. They were mostly classified as Juniors (51.1%), majored in social sciences (including anthropology, economics, and psychology; 65.5%), were never married (88.2%), and lived off-campus (94.8%) with their parents (54.6%). Table 1 includes all participants’ demographic information. Table 2 includes the correlations, means, and standard deviations of the study variables.

### 3.2. Preliminary Analyses

#### Independent Samples *T*-Test

Independent samples *t*-tests were performed to examine the differences between each eating style among those categorized with normal weight status (group #1) and overweight/obese weight status (group #2). Among the non-purposeful eating styles, there were significant differences in emotional (*t* = −2.42, *p* = 0.018) and uncontrolled eating (*t* = −2.58, *p* = 0.012) between normal and overweight/obese weight classifications. The average scores for emotional and uncontrolled eating were reduced by 7.53 (SD = 3.11) and 2.26 (SD = 0.88), respectively, compared to the overweight/obese group. The effect sizes were d = −0.48 for emotional eating and d = −0.51 for uncontrolled eating, measured by Cohen’s d, indicating an overall medium effect. Among the purposeful eating styles, intuitive eating found significant difference between the normal and overweight/obese groups (*t* = 3.12, *p* = 0.002). The average score for intuitive eating was increased by 0.38 (SD = 0.12) in the normal weight group, compared to the overweight/obese group. Effect size, measured by Cohen’s d, was d = 0.62, thus indicating a medium effect. Ad-hoc sample size estimation was confirmed using a medium effect size (0.6) and 0.8 power estimation. According to Fritz and Mackinnon (2007) [42], it was confirmed that the current study had an acceptable sample size with 104 participants [42].

### 3.3. Mediation Analyses

**Figure 3:**
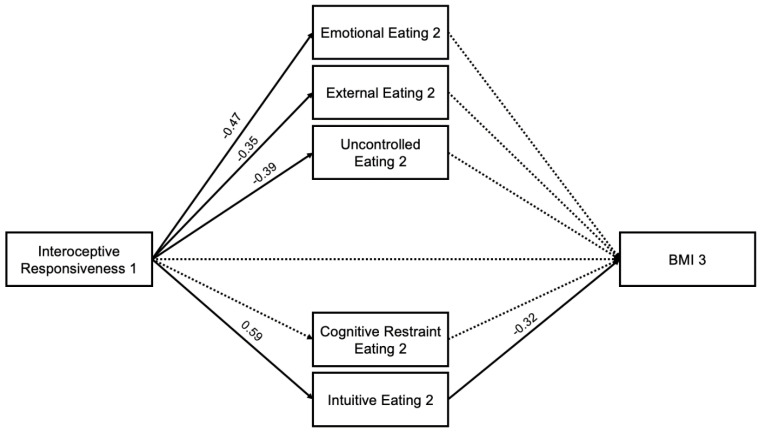
Interoceptive Responsiveness Mediation Model with Standardized Estimates. Note. Bolded lines are considered significant.

In the interoception model, increased interoceptive responsiveness (T1) was associated with reduced emotional eating (T2) (path a1 = −0.47, *p* < 0.001), external eating (T2) (path a2 = −0.35, *p* < 0.001), and uncontrolled eating (path a3 = −0.39, *p* < 0.001), and increased intuitive eating (T2) (path a5 = 0.59, *p* < 0.001). Increased intuitive eating (T2) was associated with reduced BMI (path b5 = −0.32, *p* = 0.026). There was a significant total indirect effect (−0.21, 95%CI [−0.373, −0.049]) and specific indirect effect of interoception (T1) on BMI (T3) through intuitive eating (T2) (−0.19, 95% CI [−0.394, −0.034]). All associations can be found in Figure 3.

**Figure 4:**
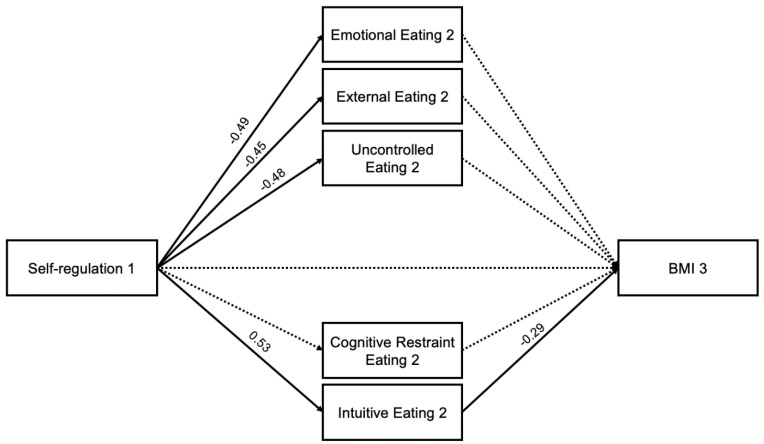
Self-regulation Mediation Model with Standardized Estimates. Note. Bolded lines are considered significant.

In the self-regulation model, increased self-regulation (T1) was associated with reduced emotional eating (T2) (path a1 = −0.49, *p* < 0.001), external eating (T2) (path a2 = −0.45, *p* < 0.001), and uncontrolled eating (path a3 = −0.48, *p* < 0.001), and increased intuitive eating (T2) (path a5 = 0.53, *p* < 0.001). Increased intuitive eating (T2) was associated with reduced BMI (path b5 = −0.29, *p* = 0.039). There was a significant specific indirect effect of self-regulation (T1) on BMI (T3) through intuitive eating (T2) (−0.15, 95%CI [−0.334, −0.026]). All associations can be found in Figure 4.

## 4. Discussion

The study’s purpose was to determine the indirect effects of specific eating styles on the relationships of interoception and self-regulation and BMI. It was hypothesized that individuals with increased interoception and self-regulation will have decreased BMI through purposeful eating (cognitive restraint/intuitive eating). Individuals with reduced interoception and self-regulation will have increased BMI through non-purposeful eating (emotional, external, uncontrolled eating). Study results indicated that intuitive eating (T2) was the only eating style that significantly mediated the relationships of interoception (T1) and self-regulation (T1) on BMI (T3), where those with increased internal regulation were more likely to have reduced BMI, through intuitive eating behaviors. In addition, a medium effect size was found among emotional, uncontrolled, and intuitive eating styles when significant differences were found among those with normal and overweight/obese weight status, indicating moderately distinct differences in eating style behaviors between groups.

To our knowledge, this study was the first to examine the relationship between internal regulation skills such as interoception and self-regulation, the 5 most common eating styles, and weight status in college women. Additionally, these associations were found within the different timepoints of a college semester, indicating directionality. The intuitive eating style significantly mediated the relationship between interoceptive responsiveness and BMI. The mind–body approach utilized in this eating style is significantly dependent on the awareness of one’s internal body signaling to guide them [47]. We theorize that those with high interoception were more responsive to their hunger/satiety cues and, therefore, were able to manage their weight more efficiently. Previously, it was found that possessing effective interoceptive awareness, where one can notice the cues from their internal signals, was a significant predictor of purposeful eating habits such as intuitive eating [8].

Study results indicated consistent findings among self-regulation and BMI through intuitive eating. College females with increased self-regulation were more likely to have reduced BMI when mediated by intuitive eating. Conceptually, self-regulation is imbedded into the subcategories of intuitive eating. This eating style has the following 4 subcategories: Eating for Physical Rather Than Emotional Reasons, Unconditional Permission to Eat, Reliance on Hunger and Satiety Cues, and Body–Food Choice Congruence [39]. Although not all subcategories were significant in previous cross-sectional analyses, most of the subcategories operate from one’s ability to self-regulate their feelings, hunger and satiety cues, and well-being [29,39,48].

Overall, there were 5 eating styles that were assessed in the study; however, emotional, external, uncontrolled, and cognitive restraint eating did not significantly mediate the relationships of interoception and self-regulation on BMI. Although the non-purposeful eating styles were all associated with reduced interoception and self-regulation, these eating behaviors did not show any sensitivity to BMI over time. For example, emotional eaters were not necessarily classified as overweight or obese by the end of the semester. It was previously found that emotional eating during positive and negative states can lead to differing intake amounts [17]. Significant differences were found between weight classifications, where the participants who were classified as overweight consumed more when feeling negative emotions; however, during positive emotional states, they consumed less [17]. Conversely, the underweight group consumed more during positive feelings and less during negative feelings [17]. Due to the intake variability among weight classifications, the relationship between interoception/self-regulation and weight status can be hard to extrapolate from this eating style. Similar findings were indicated with external eating. In a previous study among adolescents classified as external eaters, it was found that this eating style was not associated to any specific BMI trajectory [49]. It is suggested that the dysregulation of food consumption that occurs during external eating does not necessarily affect weight status. Uncontrolled eating has been commonly associated with weight gain and obesity in young adults; however, this eating style is explained as a concept based on a spectrum and includes multiple subcategories that vary in degree of severity [23]. It may be explained that specific subcategories of uncontrolled eating may relate to BMI while others do not. Future research on this theory is needed to specifically identify which subcategories correlate to BMI.

Of the purposeful eating styles, cognitive restraint did not significantly mediate the relationships between interoception and self-regulation on BMI. It is also important to note that cognitive restraint was not directly related to interoception, self-regulation, or BMI. Originally, we hypothesized that cognitive restraint was more purposeful in nature, thus significantly explaining relationship between one’s internal regulation skills and BMI. However, there are mixed results on these factors in past literature. The dieting approach behind cognitive restraint lends it to have both advantages and disadvantages. In a previous study, cognitive restraint eaters who were flexible with their eating were associated with lower BMI [50]. On the other hand, college students who participated in restrained eating were more likely to be classified as overweight or obese [51]. This can be due to the possible distinctions between successful and unsuccessful cognitive restraint, where unsuccessful dieters initially suppress their intake then later practice disinhibited eating behaviors, leading them to be unsuccessful in dietary restriction and more susceptible to obesity risk [27]. It is believed that more emphasis should be put on individuals who are deemed successful vs. unsuccessful in cognitive restraint to determine distinct outcomes with internal regulation and BMI. Overall, future research is needed to clarify the ambiguity between emotional, external, uncontrolled, and cognitive restraint eating styles and BMI among young adults.

The study had significant strengths. First, we utilized 3 timepoints within the academic term to assess potential mediation. The different timepoints of the study were compared for a more detailed look on how college student eating behavior indirectly influences the relationship of interoception and self-regulation on BMI throughout the length of a typical semester. Although, future research assessing several longitudinal timepoints is needed to effectively determine the relationship that various eating behaviors play on interoception and self-regulation on BMI in college students.

Also, it is imperative to discuss a few of the study’s limitations. First, the study utilized mediation analyses to examine the associations within interoception, self-regulation, eating behaviors, and BMI; therefore, a causal relationship between the variables cannot be considered. Also, the study included female participants only. Due to the insufficient sampling of male undergraduate students, the researchers of the study removed all males from analyses; thus, the ability to generalize the study’s results should be performed with caution. Lastly, the study assessed self-reported measures of interoception, self-regulation, eating behaviors, and BMI. It should be acknowledged that the study results are based on how the participants perceived their feelings and behaviors, rather than their physiological behavior.

## 5. Conclusions

Overall, this study aimed to determine which eating style most importantly explained the relationship of interoception and self-regulation on BMI. Intuitive eating was the only mediator that explained the associated relationships among 5 most common eating styles. We presume that both interoception and self-regulation were already practiced among college students who intuitively eat, providing it eligible to accurately explain the associations on BMI. Study results can be useful in future interventions, targeting internal regulation skills and intuitive eating approaching, in college students to aid in reducing risk for obesity. Additionally, longitudinal research studies focusing on physiological behavior are needed to examine the long-term effects of various eating styles, internal regulation skills, and weight status to determine potential predictors of increased obesity risk in this population. It is important to determine the generalizability of our findings and examine the mediating effect of eating styles on internal regulation skills and BMI in other populations and conditions. For example, the ability to self-regulate can be limited due to natural aging. Daily food intake also tends to be reduced in the elderly population. Implications for how these factors influence weight status and the prevention of obesity risk in the older adult population have yet to be determined. Similarly, individuals classified in different weight categories (e.g., overweight, obese classifications) may have differing internal regulation skills that, in turn, affect their ability to practice optimal eating styles, like intuitive eating. This can result in weight gain throughout time. More research is needed among various populations to determine our study’s replicability with similar findings in various populations.

## Figures and Tables

**Table 1:** Baseline Participant Demographic Information.

		Sample	Percentage (%)
Race	American Indian or Native American	1	0.4
	Asian or Pacific Islander	6	2.6
	Black or African American	34	15
	White or Caucasian	126	55
	Other or Mixed	62	24
Ethnicity	Hispanic	172	75
	Non-Hispanic	57	25
Classification	Freshman	11	4.8
	Sophomore	38	17
	Junior	117	51
	Senior	63	28
BMI Category	Underweight	6	2.6
	Normal	125	55
	Overweight	60	26
	Obese	38	16
College Transfer	Started here	83	36
	Transferred	146	64
Major	Biological/Life Sciences	10	4.4
	Business	5	2.2
	Communication	2	0.9
	Education	1	0.4
	Engineering	1	0.4
	Health-related fields (nursing, physical therapy)	7	3.1
	Humanities	1	0.4
	Physical sciences (physics, chemistry)	1	0.4
	Pre-professional (pre-dental, pre-medical)	5	2.2
	Public administration	3	1.3
	Social sciences (anthropology, psychology)	150	66
	Visual and performing arts	1	0.4
	Other	11	4.8
Marital Status	Never married	202	88
	Married	18	7.9
	Divorced	3	1.3
	Separated	6	2.6
Living location	On-campus housing	12	5.2
	Off-campus housing	217	95
Living arrangements	Living alone	14	6.1
	With other students	17	6.1
	My family (spouse or children)	35	14
	Parents	125	55
	Other relatives	7	3.0
	Other	7	3.0

**Table 2:** Correlations, Means, and Standard Deviations of Cognitive Skills, Eating styles, and BMI.

Variables	1	2	3	4	5	6	7	8
1. BRS	1	0.567 **	−0.365 **	−0.307 **	−0.365 **	−0.103	0.563 **	−0.246 *
2. SREBQ		1	−0.401 **	−0.350 **	−0.438 **	−0.031	0.459 **	−0.309 **
3. Emotional			1	0.575 **	0.570 **	0.225 **	−0.654 **	0.294 **
4. External				1	0.680 **	0.103	−0.377 **	0.164
5. Uncontrolled					1	0.224 **	−0.329 **	0.219 *
6. Cognitive Restraint						1	−0.225 **	0.167
7. Intuitive							1	−0.387 **
8. BMI								1
Mean	31.38	3.190	33.24	31.41	21.56	14.69	3.465	25.23
Standard deviation	7.86	0.72	15.56	7.69	4.30	2.56	0.61	4.64

Note: * Significant at 0.05 level. ** Significant at 0.01 level.

## Data Availability

The data presented in this study are available on request from the corresponding author due to the sensitive information collected from participants, the data from this study will remain confidential and unshared, as promised to our participants.
